# Stenotrophomonas maltophilia from Nepal Producing Two Novel Antibiotic Inactivating Enzymes, a Class A β-Lactamase KBL-1 and an Aminoglycoside 6′-*N*-Acetyltransferase AAC(6′)-Iap

**DOI:** 10.1128/spectrum.01143-22

**Published:** 2022-07-07

**Authors:** Ryota Kawauchi, Tatsuya Tada, Jatan B. Sherchan, Shovita Shrestha, Mari Tohya, Tomomi Hishinuma, Teruo Kirikae, Jeevan B. Sherchand

**Affiliations:** a Department of Microbiology, Juntendo University Graduate School of Medicine, Tokyo, Japan; b Department of Clinical Microbiology, Kathmandu University School of Medical Sciences, Dhulikhel, Nepal; c Department of Microbiology, Institute of Medicine, Tribhuvan University, Kathmandu, Nepal; Universidad de Buenos Aires, Facultad de Farmacia y Bioquímica

**Keywords:** *Stenotrophomonas maltophilia*, drug resistance mechanisms, KBL-1, AAC(6′)-Iap

## Abstract

Seven drug-resistant strains of Stenotrophomonas maltophilia were isolated from patients at two university hospitals in Nepal. S. maltophilia JUNP497 was found to encode a novel class A β-lactamase, KBL-1 (Kathmandu β-lactamase), consisting of 286 amino acids with 52.98% identity to PSV-1. Escherichia coli transformants expressing *bla*_KBL-1_ were less susceptible to penicillins. The recombinant KBL-1 protein efficiently hydrolyzed penicillins. The genomic environment surrounding *bla*_KBL-1_ was a unique structure, with the upstream region derived from strains in China and the downstream region from strains in India. S. maltophilia JUNP350 was found to encode a novel 6′-N-aminoglycoside acetyltransferase, AAC(6′)-Iap, consisting of 155 amino acids with 85.0% identity to AAC(6′)-Iz. E. coli transformants expressing *aac(6′)-Iap* were less susceptible to arbekacin, amikacin, dibekacin, isepamicin, neomycin, netilmicin, sisomicin and tobramycin. The recombinant AAC(6′)-Iap protein acetylated all aminoglycosides tested, except for apramycin and paromomycin. The genomic environment surrounding *aac(6′)-Iap* was 90.99% identical to that of S. maltophilia JV3 obtained from a rhizosphere in Brazil. Phylogenetic analysis based on whole genome sequences showed that most S. maltophilia isolates in Nepal were similar to those isolates in European countries, including Germany and Spain.

**IMPORTANCE** The emergence of drug-resistant S. maltophilia has become a serious problem in medical settings worldwide. The present study demonstrated that drug-resistant S. maltophilia strains in Nepal harbored novel genes encoding a class A β-lactamase, KBL-1, or a 6′-N-aminoglycoside acetyltransferase, AAC(6′)-Iap. Genetic backgrounds of most S. maltophilia strains in Nepal were similar to those in European countries. Surveillance of drug-resistant S. maltophilia in medical settings in Nepal is necessary.

## INTRODUCTION

Stenotrophomonas maltophilia is a globally emergent, multidrug-resistant Gram-negative pathogen frequently associated with respiratory tract and bloodstream infections in immunocompromised patients (1). Between 1997 and 2016, a total of 6,467 S. maltophilia isolates were reported from 259 medical settings in 43 countries worldwide, including Asia-Pacific, Latin America, Europe, and North America. These isolates were obtained from hospitalized patients with pneumonia (55.8%), bloodstream infections (33.8%), skin infections (7.8%), urinary tract infections (1.2%), and intra-abdominal infections (1.0%) (2).

The chromosome of S. maltophilia intrinsically harbors two genes, *bla*_L1_ and *bla*_L2_, that encode the two β-lactamases, L1 and L2, respectively. L1 is a broad-spectrum metallo-β-lactamase that hydrolyzes carbapenems, whereas L2 is a serine β-lactamase that hydrolyzes cephalosporins (3). S. maltophilia also harbors two sets of genes associated with intrinsic multidrug resistance, including genes encoding lytic transglycosylases (MltA, MltB1, MltB2, MltD1, MltD2, and Slt) and genes encoding an efflux system (SmeD, SmeE, and SmeF); however, these factors do not affect susceptibility to aminoglycosides, such as amikacin, gentamicin, kanamycin, streptomycin, and tobramycin (4, 5). On the other hand, evaluation of aminoglycoside resistance due to modification enzymes showed that S. maltophilia is likely to intrinsically harbor a gene, *aph (3′)-IIc*, encoding an aminoglycoside phosphotransferase enzyme that significantly decreases bacterial susceptibility to kanamycin, neomycin, butirosin, and paromomycin (6), and that most S. maltophilia isolates harbor a gene, *aac(6′)-Iz*, encoding an aminoglycoside acetyltransferase that reduces susceptibility to amikacin, netilmicin, and tobramycin (7, 8). S. maltophilia isolates also harbor two genes, *aac(6′)-Iam* and *aac(6′)-Iak*, closely related to *aac(6′)-Iz* (9, 10).

The present study describes two clinical isolates of S. maltophilia obtained from hospitalized patients in Nepal, one harboring a gene encoding a novel class A β-lactamase, KBL-1, and the other harboring a gene encoding a novel 6′-N-aminoglycoside acetyltransferase, AAC(6′)-Iap.

## RESULTS AND DISCUSSION

### Drug susceptibilities of S. maltophilia isolates.

Of the seven S. maltophilia isolates, five were resistant to ceftazidime, three were resistant to ticarcillin-clavulanic acid, and one each was resistant to chloramphenicol, levofloxacin, and sulfamethoxazole-trimethoprim (Table 1). All seven isolates had MICs of ≥64 μg/mL for imipenem, meropenem, and colistin, and six each had MICs of ≥128 μg/mL for aztreonam, arbekacin, and amikacin.

**TABLE 1:** Characteristics of the seven S. maltophilia strains in Nepal, including their antimicrobial resistance profiles and drug-resistant factors

Strain	MIC (μg/mL)^*a*^													β-lactamase		Aminoglycoside modifying enzyme(s)
	ABK	AMK	AZT	CAZ	CHL	CIP	CST	IPM	LVX	MEM	MIN	SXT	TIM	Metallo-β-lactamase	Serine β-lactamase^*b*^	
JUNP052	128	256	512	64	16	2	>128	512	2	128	0.5	2/38	128/2	L1	L2	APH(3′)-IIc
JUNP329	256	128	512	8	8	1	64	128	0.5	128	≤0.25	0.2/3.8	16/2	L1	L2	APH(3′)-IIc
JUNP349	>512	512	>512	32	16	2	>128	128	2	64	0.5	1/19	128/2	L1	L2	AAC(6′)-Iap, APH(3′)-IIc
JUNP350	>512	256	>512	32	8	2	>128	128	1	64	0.5	1/19	64/2	L1	L2	A AAC(6′)-Iap, APH(3′)-IIc
JUNP351	>512	512	>512	32	16	2	>128	512	4	256	≤0.25	1/19	64/2	L1	L2	AAC(6′)-Iak, APH(3′)-IIc
JUNP461	16	8	4	2	8	4	>512	256	0.25	128	≤0.25	0.3/4.7	32/2	L1	L2	APH(3′)-IIc
JUNP497	>512	128	>512	64	128	128	>512	256	32	64	2	8/152	128/2	L1	L2, KBL-1, PME-1	AAC(6′)-Iz, APH(3′)-IIc, APH(3′)-VI

a ABK, arbekacin; AMK, amikacin; AZT, aztreonam; CAZ, ceftazidime; IPM, imipenem; MEM, meropenem; CIP, ciprofloxacin; LVX, levofloxacin; CST, colistin; SXT, trimethoprim-sulfamethoxazole; CHL, chloramphenicol; TIM, ticarcillin-clavulanic acid; MIN, minocycline.

b ESBL, extended-spectrum β-lactamase.

### Drug resistance genes.

Assessment of β-lactamase encoding genes showed that all seven isolates harbored *bla*_L1_ and *bla*_L2_, genes intrinsic to S. maltophilia (3). In addition, one isolate, JUNP497, harbored two other genes encoding β-lactamases, *bla*_PME-1_ and *bla*_KBL-1_, a gene encoding a novel class A β-lactamase. Evaluation of genes encoding aminoglycoside modifying enzymes showed that all seven isolates harbored genes encoding APH(3′), including *aph(3′)-IIc* and *aph(3′)-IV*. In addition, four isolates harbored genes encoding AAC(6′), such as *aac(6′)-Iz*, *aac(6′)-Iak* and *aac(6′)-Iap*, a novel gene encoding an aminoglycoside acetyltransferase.

### A novel class A β-lactamase KBL-1.

**FIG 1:**
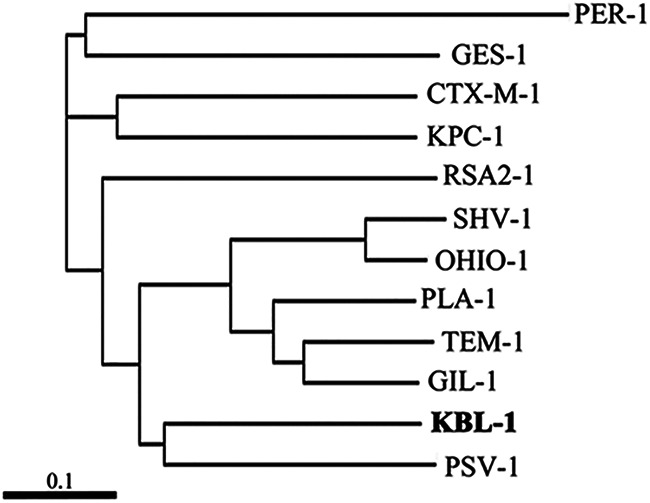
Dendrogram comparing class A β-lactamases with KBL-1. The dendrogram was calculated using the Clustal Omega program. Branch lengths correspond to numbers of amino acid exchanges among the proteins. The EMBL/GenBank/DDBJ accession numbers of the proteins are PER-1, WP_001100753; GES-1, WP_013250881; CTX-M-1, WP_013188473; KPC-1, AF297554; RSA2-1, NG_063889; SHV-1, NG_049989; OHIO-1, NG_049352; PLA-1, NG_049969; TEM-1, NG_050145; GIL-1, NG_049142; KBL-1, LC579778; PSV-1, NG_052626.

The novel class A β-lactamase KBL-1 consisted of 286 amino acids. A comparison of its sequence to the amino acid sequences of 10 representative class A β-lactamases showed that KBL-1 were closest to PSV-1, with 52.98% sequence identity (Fig. 1). PSV-1 had previously been identified in Pseudovibrio ascidiaceicola, obtained from a species of sponge, *Aplysina aerophoba*, in Spain (11). Compared with the vector control, E. coli expressing *bla*_KBL-1_ showed much higher MIC values (256 to 4,096 μg/mL) toward the penicillins, including ampicillin, amoxicillin, penicillin G, and piperacillin, with the MICs toward these penicillins being 2,048, 256, 128, and 512-fold higher, respectively, for E. coli expressing *bla*_KBL-1_ than for the vector control (Table 2). The MICs of these penicillins were significantly reduced by β-lactamase inhibitors combined with penicillins, including amoxicillin-clavulanic acid, ampicillin-sulbactam, and piperacillin-tazobactam, which had MICs of 32 to 128 μg/mL. The E. coli expressing *bla*_KBL-1_showed lower MICs for the monobactam aztreonam; the cephalosporins cefepime, cefotaxime, cefoxitin, ceftazidime, cefozopran, cephradine, and moxalactam; and the carbapenems doripenem, imipenem, meropenem, and panipenem. Moreover, except for ceftazidime, there were no significant differences in the MICs of E. coli expressing *bla*_KBL-1_ and the vector control for any of these agents. The MIC of ceftazidime for E. coli expressing *bla*_KBL-1_ was low (1 μg/mL), but significantly higher than that for the vector control (0.125 μg/mL), suggesting that measurement conditions, such as salinity, temperature, and pH, may affect the hydrolysis of ceftazidime.

**TABLE 2:** MICs of β-lactam for S. maltophilia JUNP497 and E. coli strains transformed with *bla*_KBL-1_

β-lactams	MIC(μg/mL)		
	E. coli DH5α/ pHSG398-*bla*_KBL-1_	E. coli DH5α/ pHSG398	JUNP497
Ampicillin	4,096	2	128
Amoxicillin	1,024	4	256
Aztreonam	0.063	0.063	>512
Penicillin G	2,048	16	128
Cephradine	8	8	128
Cefoxitin	4	4	16
Ceftazidime	1	0.125	64
Cefotaxime	0.031	0.031	128
Cefepime	0.016	0.016	32
Cefozopran	0.125	0.063	128
Imipenem	0.125	0.125	256
Meropenem	0.031	0.031	32
Piperacillin	256	0.5	64
Moxalactam	0.25	0.25	1
Clavulanic acid/amoxicillin	32	8	128
Sulbactam/ampicillin	128	2	128
Tazobactam/piperacillin	64	0.5	32
Panipenem	0.125	0.125	256
Doripenem	0.031	0.031	128

Recombinant KBL-1 protein had hydrolytic activities against all the β-lactams tested, except for aztreonam (Table 3). Recombinant KBL-1 efficiently hydrolyzed the penicillins, including ampicillin, amoxicillin, penicillin G, and piperacillin with *k_cat_*/*k_m_* values of 0.422 to 1.166, whereas it slightly hydrolyzed cephalosporins and carbapenems with *k_cat_*/*k_m_* values of 0.001 to 0.044. IC_50_ determinations performed with penicillin G as a substrate showed that KBL-1 activity was very well inhibited by 0.21 μM clavulanic acid and 1.2 μM sulbactam.

**TABLE 3:** Enzymatic activities of KBL-1 recombinant protein against β-lactams^*a*^

β-lactams	*K*_m_ (μM)^*b*^	*K*_cat_ (s^−1^)^*b*^	*K*_cat_*/K*_m_ (μM^−1^s^−1^)
Ampicillin	37 ± 8	19 ± 1	0.532
Amoxicillin	23 ± 4	10 ± 0.4	0.455
Aztreonam	NH^*c*^		
Penicillin G	21 ± 5	24 ± 2	1.166
Cephradine	605 ± 45	2.23 ± 0.13	0.004
Cefoxitin	57 ± 6	0.032 ± 0.002	0.001
Ceftazidime	45 ± 12	0.013 ± 0.003	0.0003
Cefotaxime	31 ± 5	1.332 ± 0.053	0.044
Cefepime	79 ± 2	0.751 ± 0.029	0.010
Imipenem	85 ± 7	1.132 ± 0.143	0.013
Meropenem	39 ± 6	0.443 ± 0.056	0.011
Piperacillin	52 ± 7	22 ± 1	0.422
Moxalactam	49 ± 4	0.392 ± 0.009	0.008

a The proteins were initially modified with a His-tag, which was removed after purification.

b *K_m_* and *k_cat_* values represent the means ± standard deviations of the results of three independent experiments.

c NH: no hydrolysis was detected under conditions with substrate concentrations up to 1 mM and enzyme concentration up to 700 nM.

### A novel 6′-*N*-aminoglycoside acetyltransferase AAC(6′)-Iap.

**FIG 2:**
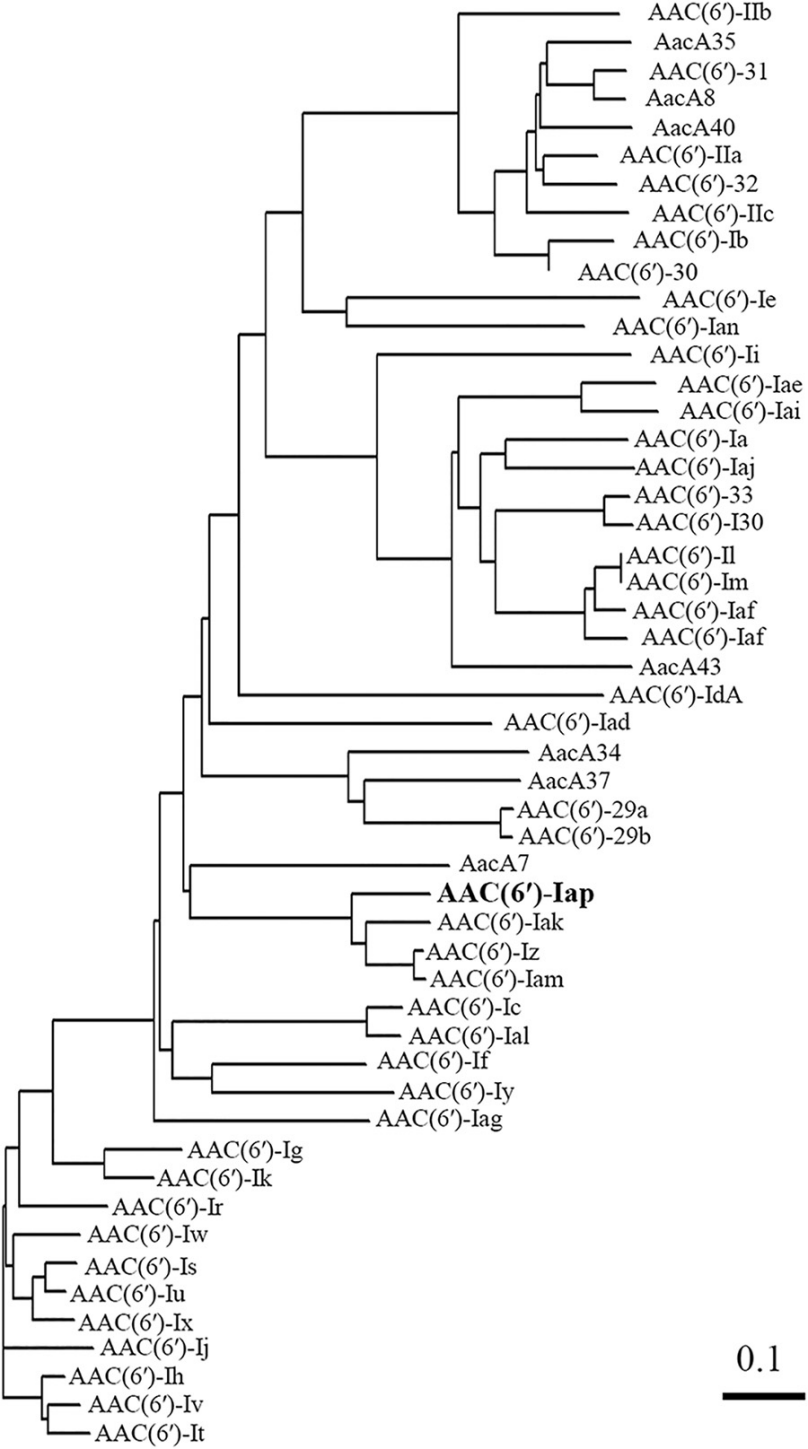
Dendrogram comparing 6′-N-aminoglycoside acetyltransferases [AAC(6′)s with AAC(6′)-Iap]. The dendrogram was calculated using the Clustal Omega program. Branch lengths correspond to numbers of amino acid exchanges among the proteins. The EMBL/GenBank/DDBJ accession numbers of the proteins are AAC(6′)-Ia, M18967-1; AAC(6′)-Ib, M23634; AAC(6′)-Ic, M94066; AAC(6′)-Id, X12618; AAC(6′)-Ie, M13771; AAC(6′)-If, X55353;AAC(6′)-Ig, L09246; AAC(6′)-Ih, L29044; AAC(6′)-Ii, L12710-1; AAC(6′)-Ij, L29045; AAC(6′)-Ik, L29510; AAC(6′)-Il, U13880; AAC(6′)-Im, AF337947; AAC(6′)-Iq, AF047556-1; AAC(6′)-Ir, AF031326; AAC(6′)-Is, AF031327; AAC(6′)-It, AF031328; AAC(6′)-Iu, AF031329; AAC(6′)-Iv, AF031330; AAC(6′)-Iw, AF031331; AAC(6′)-Ix, AF031332; AAC(6′)-Iy, AF144880; AAC(6′)-Iz, AF140221; AAC(6′)-Iad, AB119105; AAC(6′)-Iae, AB104852; AAC(6′)-Iaf, AB462903; AAC(6′)-Iag, AB472901; AAC(6′)-Iai, EU886977; AAC(6′)-Iaj, AB709942; AAC(6′)-Iak, AB894482; AAC(6′)-Ial, AB871481; AAC(6′)-Iam, AB971834; AAC(6′)-Ian, NG_047282; AAC(6′)-Iap, LC536747; AAC(6′)-IIa, M29695; AAC(6′)-IIb, L06163; AAC(6′)-IIc, AF162771; AAC(6′)-29a, AF263519; AAC(6′)-29b, AF263519; AAC(6′)-30, AJ584652; AAC(6′)-31, AJ640197; AAC(6′)-32, EF614235; AAC(6′)-33, GQ337064; AAC(6′)-I30, AY289608; AacA7, U13880; AacA8, AY444814; AacA34, AY553333; AacA35, AJ628983; AacA37, DQ302723; AacA40, EU912537; and AacA43, HQ247816.

**FIG 3:**
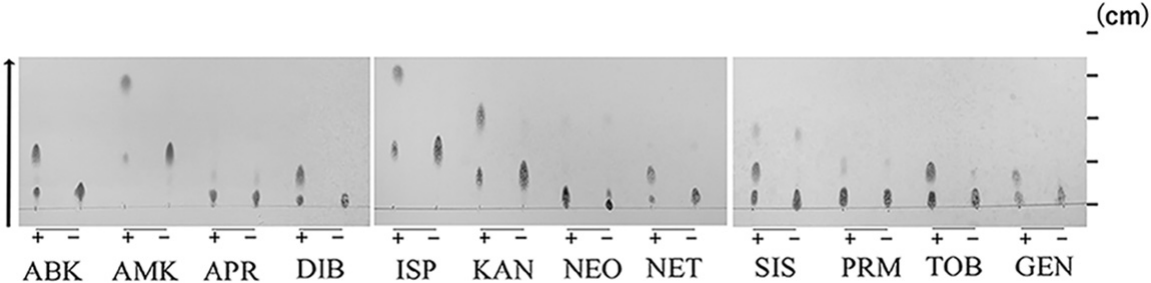
Analysis of acetylated aminoglycosides by thin-layer chromatography. AAC(6′)-Iap and various aminoglycosides were incubated in the presence (+) or absence (-) of acetyl coenzyme A. ABK, arbekacin; AMK, amikacin; APR; apramycin; DIB, dibekacin; ISP, isopamicin; KAN, kanamycin; NEO, neomycin; NET, netilmicin; SIS, sisomicin; PRM, paromomycin; TOB, tobramycin; GEN, gentamicin.

The AAC(6′)-Iap protein was found to consist of 155 amino acids. Multiple sequence alignments among AAC(6′) enzymes revealed that AAC(6′)-Iap was 85.0% identical to AAC(6′)-Iz (7), 83.0% identical to AAC(6′)-Iam (9), and 79.1% identical to AAC(6′)-Iak (10) (Fig. 2). Compared with vector control, E. coli expressing AAC(6′)-Iap showed decreased susceptibilities to arbekacin, amikacin, dibekacin, isepamicin, neomycin, netilmicin, sisomicin, and tobramycin (Table 4). Thin-layer chromatography (TLC) analysis revealed that all the aminoglycosides tested, except for apramycin and paromomycin, were acetylated by AAC(6′)-Iap (Fig. 3). These results indicated that *aac(6′)-Iap* is a functional acetyltransferase that modifies the 6′-NH_2_ position of aminoglycosides and is involved in aminoglycoside resistance. The TLC data for apramycin and paromomycin were consistent with the MICs of the aminoglycosides for E. coli with pSTV28-*aac(6′)-Iap*. Although gentamicin and kanamycin were acetylated by AAC(6′)-Iap, the MICs were not higher than those of E. coli harboring pSTV28-*aac(6′)-Iap.* Gentamicin includes gentamicins C1, C2, and C1a, with gentamicin C1 having no amino group at the 6′-position, suggesting that gentamicin may only have been partially acetylated by AAC(6′)-Iap.

**TABLE 4:** MICs of aminoglycosides for S. maltophilia JUNP350 and E. coli strains transformed with *aac(6′)-Iap*^*a*^

Strain	MIC (μg/mL)^*b*^											
	ABK	AMK	APR	DIB	GEN	ISP	KAN	NEO	NET	SIS	TOB	PRM
S. maltophilia JUNP350	>512	256	512	512	16	16	16	128	256	256	128	32
E. coli DH5α/pSTV28	≤0.25	1	2	≤0.25	≤0.25	≤0.25	1	0.5	≤0.25	≤0.25	≤0.25	1
E. coli DH5α/pSTV28*-aac(6′)-Iap*	8	2	2	4	≤0.25	0.5	2	1	0.5	0.5	2	1

a The MICs for S. maltophilia and E. coli strains were determined with Mueller-Hinton broth preparations and individual aminoglycosides.

b ABK, arbekacin; AMK, amikacin; APR, apramycin; DIB, dibekacin; GEN, gentamicin; ISP, isepamicin; KAN, kanamycin; NEO, neomycin; NET, netilmicin; SIS, sisomicin; TOB, tobramycin; PRM, paromomycin.

### Genomic environments surrounding *bla*_KBL-1_ and *aac(6′)-Iap*.

**FIG 4:**
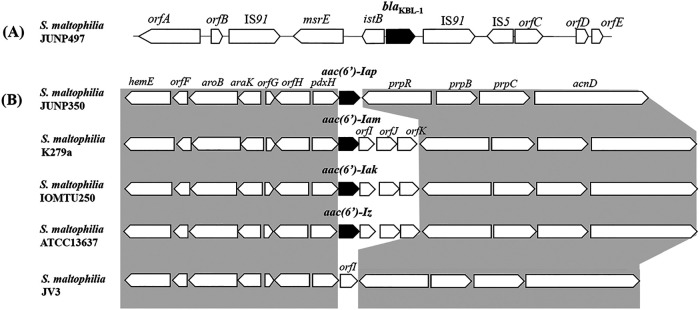
Genetic environments surrounding (A) *bla*_KBL-1_ and (B) *aac(6′)-Iap*, both of which were located on the S. maltophilia chromosome. (A) The *bla*_KBL-1_ surrounding region, *msrE-istB-bla*_KBL-1_, was flanked by IS*91*. *orfA*, ATP-binding protein encoding gene; *orfB*, phosphoglucosamide mutase encoding gene; and *orfC*, *D and E*, hypothetical proteins encoding genes. (B) The genetic environment surrounding *aac(6′)-Iap* was similar to that of S. maltophilia JV3 obtained in Brazil (GenBank accession no. CP050452). *orfF*, WGR domain-containing protein encoding gene; *orfG*, dodecin family protein encoding gene; *orfH*, d-glycerate 3-kinase encoding gene; *orfI*, hypothetical protein encoding gene; *orfJ*, DoxX family protein encoding gene; *orfK*, SMI1/KNR4 family protein encoding gene.

The genomic environment surrounding *bla*_KBL-1_ was a unique structure, consisting of *orfA-orfB-*IS*91*-*msrE*-*istB*-*bla*_KBL-1_-IS*91*-IS*5*-*orfC*-*orfD*-*orfE* (Fig. 4A). The *bla*_KBL-1_ surrounding region, *msrE*-*istB*-*bla*_KBL-1_, was flanked by IS*91*. BLAST analysis did not identify any similar structure in GenBank, suggesting that this structure may be unique.

The genomic environment surrounding *aac(6′)-Iap* consisted of *hemE-orfF*-*aroB*-*aroK-orfG-orfH*-*pdxH-aac(6′)-Iap*-*prpR*-*prpB*-*prpC*-*acnD*, which was 90.99% (nucleotides [nt] 3368404 to 3374930; GenBank accession no. CP002986) identical to a strain of S. maltophilia JV3 obtained from the rhizosphere in Brazil and 90.17% (nt 3279480 to 3286016; GenBank accession no. CP050452) identical to a strain of S. maltophilia SoD9b obtained in the Collins Glacier beach area in Antarctica (12). The upstream (*hemE-orfF*-*aroB*-*aroK-orfG-orfH*-*pdxH*) and downstream (*prpR*-*prpB*-*prpC*-*acnD*) regions of not *aac(6′)-Iap* were identical to those in S. maltophilia K279a harboring *aac(6′)-Iam* (9), IOMTU250 harboring *aac(6′)-Iak* (10). and ATCC13637 harboring *aac(6′)-Iz* (7) and JV3 (accession no. CP002986) (Fig. 4B). These results suggested that the genetic structures surrounding *aac(6′)-Iap* are widely conserved among S. maltophilia samples obtained in various countries.

BLAST analysis revealed that the genetic structure surrounding *aac(6′)-Iap*, *pdsX-aac(6′)-Iap-prpR*, was identical to the structures in environmentally arising S. maltophilia strains. Most of these strains had been obtained from environmental sources, including a laboratory sink, river water, soil, and wastewater (Table S1). In contrast, the genetic structures surrounding *aac(6′)-Iam/-Iak/-Iz*, *pdxH-aac(6′)-Iam/-Iak/-Iz-orfI-orfJ-orfK* were identical to those in S. maltophilia strains obtained from medical settings. The genomic structure surrounding *aac(6′)-Iap* seems to be new, combining structures detected in medical settings and in environmental sources.

### Phylogenetic analysis of S. maltophilia in Nepal.

**FIG 5:**
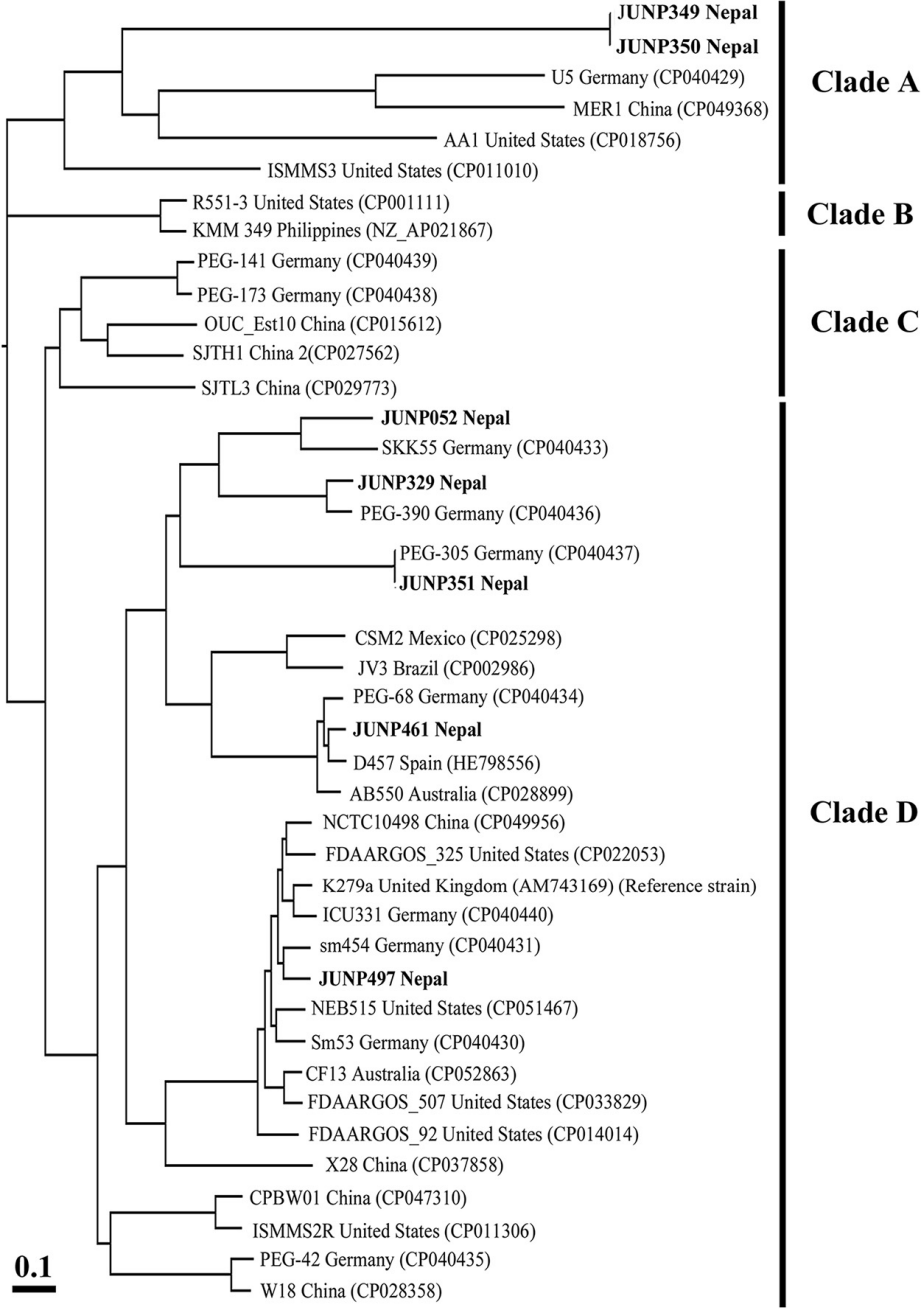
Molecular phylogenetic tree of S. maltophilia strains based on the whole genome sequences of the seven S. maltophilia strains obtained in Nepal (shown in bold) and 35 S. maltophilia strains obtained in various countries, including Australia, Brazil, China, Filipin, Germany, India, Mexico, Spain, and the United States, and registered in GenBank.

Phylogenetic analysis based on whole genome sequences revealed that S. maltophilia can be divided into three clades, designated A, B, C and D (Fig. 5). Clade A consisted of the isolates obtained in Nepal in 2019 (JUNP349 and JUNP350), in China in 2019, in Germany in 2018, and in the United States in 2012 and 2013. The SNPs between JUNP349 and JUNP350 were 157. Clade B consisted of the isolates obtained in the Philippines in 1991 and the unknown strain R551-3. Clade D consisted of the isolates obtained in Nepal in 2012. Clade C consisted of the isolates obtained in China in 2013 and 2017, and in Germany in 2018. Clade D consisted of the isolates obtained in Nepal in 2012, 2018 (JUNP052), and 2019 (JUNP351, JUNP461, JUNP329, and JUNP497); in Australia in 2011 and 2016; in Brazil in 2011; in China in 2012, 2016, 2017, and 2019; in Germany in 2018; in India in 1964; in Mexico in 2016; in Spain in 2013; and in the United States in 2013 and 2015; and the unknown strain NEB515.

Most S. maltophilia clinical isolates in Nepal were derived from strains in European countries, including Germany and Spain, whereas the S. maltophilia strains JUNP349 and JUNP350 were indigenous to Nepal. Based on SNPs, the genetic backgrounds of the two clonal strains described in this study differed from those of other strains. The present study suggests that most S. maltophilia strains obtained in Nepal had similar genetic background to the wide-distributed strains belonging to Clade D. In several countries, including Australia, Brazil, Germany, Mexico, and the United States, where the wide-distributed strains belonging to Clade D were isolated, the isolation rates of levofloxacin-resistant S. maltophilia were relatively high, according to the SENTRY Antimicrobial Surveillance Program (1997–2016) (2). It is important to continue antimicrobial surveillance of S. maltophilia in Nepal and analyze the genetic backgrounds.

## MATERIALS AND METHODS

### Bacterial strains.

Between April 2018 and November 2019, seven S. maltophilia isolates were obtained from seven patients treated at two hospitals in Kathmandu, Nepal (six isolates from hospital A and one from hospital B). The bacteria were identified using the biochemical API 20 NE test (bio-Mérieux, Marcy L'Etoile, France) and by sequencing their 16S rRNA genes. Of the seven isolates, three were from respiratory tracts, two from pus, one from blood, and one from cerebrospinal fluid. Escherichia coli DH5α (TaKaRa Bio, Shiga, Japan) and E. coli BL21-CodonPlus (DE3)-RIP (Agilent Technologies, Santa Clara, CA) were used as hosts for recombinant plasmids and protein expression, respectively. MICs were determined using a broth microdilution method, with the breakpoints of ceftazidime, chloramphenicol, levofloxacin, minocycline, ticarcillin-clavulanic acid, and trimethoprim-sulfamethoxazole for S. maltophilia determined according to the guidelines of the Clinical and Laboratory Standards Institute (13).

### Whole-genome sequencing.

Genomic DNA was extracted from each of the seven isolates using DNeasy blood and tissue kits (Qiagen, Tokyo, Japan) and sequenced using the MiSeq platform (Illumina, San Diego, CA) with the Nextera XT DNA library prep kit and MiSeq reagent kit version 3 (600 cycle; Illumina). More than 30-fold coverage was achieved for each isolate. Raw reads of each isolate were assembled using CLC Genomics Workbench version 10.0.1, and drug-resistant genes were identified using ResFinder 3.0 (https://cge.food.dtu.dk/services/ResFinder/). Fluoroquinolone resistance has been associated with mutations in the quinolone resistance-determining region, which includes the *gyrA* and *parC* genes that encode DNA gyrase and topoisomerase IV, respectively (14).

### Phylogenetic analysis based on single nucleotide polymorphisms (SNPs).

The complete genome sequences of 35 isolates of S. maltophilia isolates obtained in various countries were collected from GenBank (https://www.ncbi.nlm.nih.gov/nuccore). These sequences were aligned against the sequence of S. maltophilia K279a isolated in the United Kingdom in 1998 (GenBank accession no. AM743169), and a phylogenetic tree was constructed using kSNP3.0.

### Escherichia coli transformants expressing *bla*_KBL-1_ and *aac(6′)-Iap*.

The open reading frame of *bla*_KBL-1_ was PCR amplified using the primers EcoRI-KBL-1-1F (5′-ATGAATTCATGCGTCTTACATTTCCCTTCG-3′) and PstI-KBL-1-R (5′- ATCTGCAGTTAGCGCCTTGCTTGGATTTCG-3′). The open reading frame of *aac(6′)-Iap* and its promoter region was PCR amplified using the primers EcoRI-AAC(6′)-Iap-F (5′-ATGAATTCAGTCGAAGACGCTTGCAACGCG -3′) and BamHI-AAC(6′)-Iap-R (5′-ATGGATCCCTACCCCCGCGTGACCGCGTCC -3′). E. coli transformants expressing *bla*_KBL-1_ and *aac(6′)-Iap* were produced as previously described (10). The PCR product of *bla*_KBL-1_ was digested with EcoRI and PstI and ligated into pHSG398 (TaKaRa Bio), whereas the PCR product of *aac(6′)-Iap* was digested with EcoRI and BamHI and ligated into pSTV28 (TaKaRa Bio). E. coli DH5α was transformed with each plasmid, and the transformants were selected on Luria-Bertani agar containing 30 μg/mL chloramphenicol. The susceptibilities of these transformants to various β-lactams and aminoglycosides were assayed.

### Purification of AAC(6′)-Iap and KBL-1.

The open reading frames of KBL-1 and AAC(6′)-Iap without signal peptide regions were cloned into pET28a expression vector (Novagen, Inc., Madison, WI, USA) with the primer sets EcoRI-KBL-F (5′-ATGAATTCGAAAACCTGTATTTCCAAGGCGAAGCCGCCCGCGCGCTCGAGG-3′) and SalI-KBL-R (5′-ATGTCGACTTAGCGCCTTGCTTGGATTTCG-3′) for KBL-1, and BamHI-AAC(6′)-Iap-F (5′-ATGGATCCGAAAACCTGTATTTCCAAGGCGCTGCATGGTCGCAGCTGCGCGTGGGCCTG-3′) and XhoI-AAC(6′)Iap-R (5′-ATCTCGAGCTACCCCCGCGTGACCGCGTCCAGCGGCAT-3′) for AAC(6′)-Iap. E. coli BL21-CodonPlus (DE3)-RIP (Agilent Technologies, Santa Clara, CA) was transformed with each of these plasmids, and the recombinant KBL-1 and AAC(6′)-Iap proteins were purified using Ni-NTA agarose (Qiagen) according to the manufacturer’s instructions. His-tags were removed by digestion with TurboTEV protease (Accelagen, San Diego, CA, USA), and untagged proteins were purified by an additional passage over the Ni-NA agaroseX. The purities of the recombinant KBL-1 and AAC(6′)-Iap, as determined by SDS-PAGE, were each over 90%.

### Catalytic activities of KBL-1 recombinant protein.

The β-lactamase activities were monitored during the purification process using nitrocefin (Oxoid, Ltd., Basingstoke, United Kingdom). The initial rates of hydrolysis were determined at 37°C in 50 mM Tris-HCl (pH 7.4), 0.3 M NaCl buffer by UV-visible spectrophotometry (V-730; Jasco, Tokyo, Japan). Reactions were initiated by direct addition of substrate into the cuvettes of the spectrophotometer, allowing the UV absorption of the reaction mixture to be determined during the initial phase of the reaction. The *Km*, *kcat*, and *kcat/Km* ratios of β-lactam hydrolysis were determined from Lineweaver-Burk plots of triplicate analyses. Fifty percent inhibitory concentrations (IC_50_s) were determined for clavulanic acid and sulbactam. Various concentrations of these inhibitors were preincubated with the purified enzyme for 3 min at 30°C to determine the concentrations that reduced the hydrolysis rate of 100 μM penicillin G by 50%.

### Thin layer chromatography (TLC) analysis of acetylated aminoglycosides.

Mixtures containing 2 mM aminoglycoside, 2 mM acetyl coenzyme A (acetyl-CoA), and 50 μg/mL AAC(6′)-Iap in 20 μL phosphate buffer (pH 7.4) were incubated for 16 h at 37°C. Aliquots of 3 μL of each aminoglycoside mixture were spotted onto the surface of a Silica Gel 60 thin-layer chromatography (TLC) plate (Merck, Darmstadt, Germany), followed by development with a 5% phosphate potassium solution. The aminoglycosides and their acetylated products were detected by spraying the plates with 0.2% ninhydrin in acetone.

## References

[1] Brooke JS. 2012. *Stenotrophomonas maltophilia*: an emerging global opportunistic pathogen. Clin Microbiol Rev 25:2–41. doi:10.1128/CMR.00019-11. 22232370 PMC3255966

[2] Gales AC, Seifert H, Gur D, Castanheira M, Jones RN, Sader HS. 2019. Antimicrobial susceptibility of *Acinetobacter calcoaceticus*–*Acinetobacter baumannii* complex and *Stenotrophomonas maltophilia* clinical isolates: results from the SENTRY Antimicrobial Surveillance Program (1997–2016). Open Forum Infect Dis 6:S34–S46. doi:10.1093/ofid/ofy293. 30895213 PMC6419908

[3] Akova M, Bonfiglio G, Livermore DM. 1991. Susceptibility to β-lactam antibiotics of mutant strains of *Xanthomonas maltophilia* with high- and low-level constitutive expression of L1 and L2 β-lactamases. J Med Microbiol 35:208–213. doi:10.1099/00222615-35-4-208. 1941990

[4] Wu CJ, Huang YW, Lin YT, Yang TC. 2016. Inactivation of lytic transglycosylases increases susceptibility to aminoglycosides and macrolides by altering the outer membrane permeability of *Stenotrophomonas maltophilia*. Antimicrob Agents Chemother 60:3236–3239. doi:10.1128/AAC.03026-15. 26976867 PMC4862486

[5] Zhang L, Li XZ, Poole K. 2001. SmeDEF multidrug efflux pump contributes to intrinsic multidrug resistance in *Stenotrophomonas maltophilia*. Antimicrob Agents Chemother 45:3497–3503. doi:10.1128/AAC.45.12.3497-3503.2001. 11709330 PMC90859

[6] Okazaki A, Avison MB. 2007. Aph(3')-IIc, an aminoglycoside resistance determinant from *Stenotrophomonas maltophilia*. Antimicrob Agents Chemother 51:359–360. doi:10.1128/AAC.00795-06. 17088477 PMC1797691

[7] Lambert T, Ploy MC, Denis F, Courvalin P. 1999. Characterization of the chromosomal *aac(6')-Iz* gene of *Stenotrophomonas maltophilia*. Antimicrob Agents Chemother 43:2366–2371. doi:10.1128/AAC.43.10.2366. 10508008 PMC89484

[8] Li XZ, Zhang L, McKay GA, Poole K. 2003. Role of the acetyltransferase AAC(6')-Iz modifying enzyme in aminoglycoside resistance in *Stenotrophomonas maltophilia*. J Antimicrob Chemother 51:803–811. doi:10.1093/jac/dkg148. 12654758

[9] Crossman LC, Gould VC, Dow JM, Vernikos GS, Okazaki A, Sebaihia M, Saunders D, Arrowsmith C, Carver T, Peters N, Adlem E, Kerhornou A, Lord A, Murphy L, Seeger K, Squares R, Rutter S, Quail MA, Rajandream MA, Harris D, Churcher C, Bentley SD, Parkhill J, Thomson NR, Avison MB. 2008. The complete genome, comparative and functional analysis of *Stenotrophomonas maltophilia* reveals an organism heavily shielded by drug resistance determinants. Genome Biol 9:R74. doi:10.1186/gb-2008-9-4-r74. 18419807 PMC2643945

[10] Tada T, Miyoshi-Akiyama T, Dahal RK, Mishra SK, Shimada K, Ohara H, Kirikae T, Pokhrel BM. 2014. Identification of a novel 6'-N-aminoglycoside acetyltransferase, AAC(6')-Iak, from a multidrug-resistant clinical isolate of *Stenotrophomonas maltophilia*. Antimicrob Agents Chemother 58:6324–6327. doi:10.1128/AAC.03354-14. 25092711 PMC4187913

[11] Versluis D, Rodriguez de Evgrafov M, Sommer MO, Sipkema D, Smidt H, van Passel MW. 2016. Sponge microbiota are a reservoir of functional antibiotic resistance genes. Front Microbiol 7:1848. 27909433 10.3389/fmicb.2016.01848 PMC5112248

[12] Nunez-Montero K, Quezada-Solis D, Khalil ZG, Capon RJ, Andreote FD, Barrientos L. 2020. Genomic and metabolomic analysis of Antarctic bacteria revealed culture and elicitation conditions for the production of antimicrobial compounds. Biomolecules 10:673. doi:10.3390/biom10050673. PMC7277857 32349314

[13] Clinical and Laboratory Standards Institute. 2020. Performance standards for antimicrobial susceptibility testing: 30th informational supplement. Clinical and Laboratory Standards Institute, Wayne, PA.

[14] Nakano M, Deguchi T, Kawamura T, Yasuda M, Kimura M, Okano Y, Kawada Y. 1997. Mutations in the *gyrA* and *parC* genes in fluoroquinolone-resistant clinical isolates of *Pseudomonas aeruginosa*. Antimicrob Agents Chemother 41:2289–2291. doi:10.1128/AAC.41.10.2289. 9333065 PMC164110

